# Physicochemical Properties of MTA and Portland Cement after Addition of Aloe Vera 

**DOI:** 10.22037/iej.v12i3.10635

**Published:** 2017

**Authors:** Alvaro Henrique Borges, Orlando Aguirre Guedes, Luiz Evaristo Ricci Volpato, Gilberto Siebert Filho, Alexandre Meireles Borba, Omar Zina, Evandro Piva, Carlos Estrela

**Affiliations:** a *Department of Endodontics, University of Cuiaba, Brazil; *; b *Department of Endodontics, Federal University of Goiás, Brazil; *; c *Department of Restorative Dentistry, Federal University of Pelotas, Brazil*

**Keywords:** Aloe Vera, MTA, Physicochemical Properties, Portland Cement

## Abstract

**Introduction:**

The aim of this *in vitro* study was to determine the liquid-powder ratio, setting time, solubility, dimensional change, pH, and radiopacity of white structural and non-structural Portland cement, ProRoot MTA and MTA Bio, associated with a 2% glycolic solution containing Aloe Vera, as vehicle.

**Methods and Materials:**

Five samples of each material were used for each test, according to the American National Standards Institute/American Dental Association (ANSI/ADA) specification No. 57. Statistical analyses were performed using ANOVA and Tukey’s test at 5% significance. When sample distribution was not normal, non-parametric analysis of variance and the Kruskal-Wallis test were used (*α*=0.05).

**Results:**

No statistical differences were found in liquid-powder ratios among the tested materials. ProRoot MTA showed the longest setting time. Dimensional change values were acceptable in all groups. Also, no significant differences were found in pH values and pH was alkaline in all samples throughout the experiment. Mean radiopacity results obtained for white Portland cements did not meet ANSI/ADA requirements, and were significantly lower than those obtained for MTA-based cements. Finally, Portland cements showed significantly higher mean solubility values compared to the other samples.

**Conclusion:**

The physicochemical properties of the tested materials in association with Aloe Vera were compatible with ANSI/ADA requirements, except for the white Portland cements, which failed to meet the radiopacity specification.

## Introduction

The main compounds of mineral trioxide aggregate (MTA) are tricalcium silicate, tricalcium aluminate, tricalcium oxide, and silicate oxide [1, 2]. According to the patent, MTA is basically composed of ordinary type 1 Portland cement (PC) and bismuth oxide which improve radiopacity [3-6]. Currently, many forms of MTA have been commercially available for endodontic applications after introduction of the pioneer ProRoot MTA (Dentsply Tulsa Dental, Tulsa, OK, USA). MTA Bio (Angelus Indústria de Produtos Odontológicos Ltda, Londrina, PR, Brazil) is one of these preparations. While ProRoot MTA consists of 50-65% of calcium oxide and 15-25% of silicon dioxide [5, 7], MTA Bio, is composed of 80% of PC and 20% of bismuth oxide [8-10]. Both materials, MTA and PC, are available in white and gray formulations, according to the presence of iron oxide [5, 11, 12]. Also, depending on the presence of carbonates which are added to improve resistance, white PCs are classified as structural and non-structural formulations [13].

The chemical similarity between MTA and PC has been widely investigated. Except for the presence of bismuth oxide in MTA, the two materials share similar components [5, 7, 10, 12, 14]. Furthermore, MTA and PC have been shown to present similar antimicrobial activity [3], biocompatibility [5], sealing ability [1, 2], marginal adaptation [15], dimensional stability [8], and moisture tolerance [16]. In addition, both MTA and PC stimulate periradicular tissue healing [17] and dentin barrier formation [18]. These similarities have motivated research on the use of PC as a replacement for MTA [6].

Even though MTA has many favorable properties, there have been concerns about its elevated cost, which poses difficulties to the use of this material at all levels of health care [10]. Other shortcomings of the clinical use of MTA include long setting time and difficult handling [5, 10, 12, 19]. The manufacturer recommends mixing MTA with sterile water, which produces a grainy, sandy mixture that takes 2 h and 45 min to completely set [2] and is usually difficult to deliver to the required site and to compact adequately [19]. In this sense, Holland *et al.* [20] stated that it would be of interest to investigate vehicles other than water and saline, which could potentially facilitate the use of MTA.

Recently, the use of natural products in dentistry has grown substantially, largely due to undesirable properties of the materials classically employed. Among these products, glycolic solutions containing Aloe Vera have been increasingly investigated due to their therapeutic properties. Aloe Vera, a cactus plant that belongs to the *Liliaceae *family, has been extensively used in the pharmaceutical industry due its anti-inflammatory, antibacterial, antioxidant, antiviral, and antifungal actions, as well as for its hypoglycemic effects [21-23]. Potential dental applications of Aloe Vera have also been explored, *e.g.*, in cases of denture-related stomatitis, aphthous ulcers, angular cheilitis, and to reduce bleeding, inflammation and swelling in periodontal disease [22]. In endodontic therapy, Aloe Vera has been used as intracanal dressing and also for decontaminating gutta-percha cones [24, 25]. The effects of Aloe Vera on the physicochemical properties of MTA and PC have not been evaluated.

Therefore, the purpose of this *in vitro *study was to determine the liquid-powder ratio, setting time, solubility, dimensional change, hydrogenionic potential (pH), and radiopacity of white structural and non-structural PCs, ProRoot MTA, and MTA Bio associated with a glycolic solution containing Aloe Vera used as vehicle, taking into consideration the American National Standards Institute and American Dental Association (ANSI/ADA) specifications [26].

## Materials and Methods

The materials used in this study are described in table 1. Initially, different concentrations of glycolic solution containing Aloe Vera were tested to observe which one presented the best handling conditions for the current application, and the 2% glycolic solution was chosen. Aloe Vera was obtained from a manipulation pharmacy (Phloracea, Farm Manip. Ltda., Cuiabá, MT, Brazil). 

Liquid-powder ratio was established by weighing 3 g of the tested cement and mixing it with 0.20 mL of 2% glycolic solution containing Aloe Vera. This procedure was repeated five times for each material. The association of ProRoot MTA with distilled water, used as control, was made according to the manufacturer’s instructions, *i.e.*, at a 3:1 ratio.

For assessment of setting time, five stainless steel molds with 10 mm inner diameters and 2 mm uniform thickness were fabricated for each tested material. The cement was mixed and placed in the metallic molds. After 120±10 sec from the beginning of the mixture, the set formed by the glass plaque/metallic mold/cement was placed in a hermetically sealed plastic container kept at a constant temperature of 37±2^o^C and 95±5% air humidity, inside an incubator (Olidef Ind. e Com. Aparelhos Hospitalares, Ribeirão Preto, SP, Brazil), until the end of the test. After 150±10 sec from the beginning of the mixture, a Gilmore needle (100±0.5 g and 2±0.1 mm active tip) was vertically pressed against the horizontal surface of the material to observe indentations. This procedure was repeated at regular intervals of 60 sec until no more indentations could be observed on the cement surface. Setting time was defined as the time elapsed from the beginning of the mixture until the time when no more indentations were visible on the cement surface.

The solubility and dimensional change tests were performed in accordance with ANSI/ADA specification no. 57 [26]. The same samples used in the solubility test were employed for pH analysis. Medium (distilled water) pH was measured using a pH meter (Corning Inc., New York, USA) at different time points: 1, 3, 5, 15, and 30 min; 1, 2, 3, 4, 6, 9, 12, 24, 48, and 72 h; 4, 6, 7, 15, and 30 days after spatulation. Throughout the experiment, pH was analyzed for each sample always in the same plastic container, with no medium replacement. Five measurements were obtained for each material, at each time point. For radiopacity evaluation, the Digora^TM^ system (Soredex, Orion Corporation Ltd., Helsinki, Finland) was used. The sensor was exposed and then inserted into the laser optical reader of Digora^TM^ for Windows 5.1 software. The same phosphor plate was used for all exposures. Mean values and standard deviations were recorded for all measurements.

***Statistical analysis***

Statistical analyses were performed using analysis of variance (ANOVA) and Tukey’s test. The level of significance was set at 0.05. When sample distribution was not normal, non-parametric analysis of variance and the Kruskal-Wallis test were used (*α*=0.05). All tests were performed using the Statistical Package for the Social Sciences (SPSS) for Windows version 12.0.1 (SPSS Inc., Chicago, IL, USA).

## Results

The results obtained for each of the variables assessed are presented in table 2. 

No statistical differences were observed in liquid-powder ratio results among the cements tested. MTA Bio presented the lowest setting time value (*P*<0.05), while ProRoot MTA showed the highest (*P*<0.05). No differences in setting time were observed between white structural and non-structural PCs (*P*>0.05), although these results were significantly different from those of the other groups (*P*<0.05). 

With regard to solubility, white structural and non-structural PC were similar among themselves but both showed higher values compared to others groups (*P*>0.05). MTA Bio showed the lowest solubility value (*P*<0.05). In the dimensional change analysis, the lowest value was associated with ProRoot MTA (*P*<0.05), and white non-structural PC showed the highest value. pH values were statistically similar across all groups (*P*>0.05), *i.e.*, all the materials showed alkaline media, from the beginning to the end of the tests. Finally, the radiopacity test showed that the MTA Bio and ProRoot MTA cements showed radiopacity above 3mm of aluminum, as recommended, whereas PCs presented values below 3 mm.

## Discussion

The physicochemical properties of MTA and PC have been exhaustively studied. For instance, it has been demonstrated that MTA mixed with water presents short working time, delayed setting, and poor consistency [5, 10, 12, 19]. As a result, different materials have been used in an attempt to improve the handling and setting time properties of MTA and to facilitate its clinical use [19, 20, 27-29]. The present study evaluated the effect of using 2% glycolic solution containing Aloe Vera as vehicle on liquid-powder ratio, setting time, solubility, pH, and radiopacity of MTA-based and Portland cements.

Kogan *et al.* [19] investigated the influence of various additives (saline, 2% lidocaine, 3% NaOCl gel, chlorhexidine gluconate gel, K-Y Jelly, 3% and 5% CaCl_2_) on the setting properties of MTA and observed shorter setting time and superior handling properties with NaOCl gel. For those authors, NaOCl gel could be potentially used with MTA in a single visit scenario where an additional barrier to protect the MTA is not required. Wiltbank *et al.* [29] added classic PC accelerators (calcium chloride, calcium nitrite/nitrate, and calcium formate) to gray and white MTA and PC and observed that all three additives significantly accelerated the setting reaction of the tested materials. AlAnezi *et al.* [27] found that mixing KY liquid, CaCl_2_, and NaOCl with gray MTA improved handling properties, decreased setting time and allowed osteoblast and fibroblast attachment and spread at rates similar to those obtained with gray MTA mixed with water. 

Holland *et al.* [20] investigated the influence of type of vehicle (distilled water and propylene glycol) on apical tissue response in dog’s teeth after root canal filling with MTA at two different obturation limits. The results showed that MTA pastes prepared with either distilled water or propylene glycol as vehicles had similar biological behaviors. Duarte *et al.* [28] also evaluated the influence of propylene glycol on the chemical properties of white MTA. The results revealed that using propylene glycol as a liquid vehicle for MTA increased setting time, improved flowability, and increased pH and calcium ions release at the initial periods. In the present study, the mixture of 2% glycolic solution containing Aloe Vera with MTA and PC cements resulted in a material with putty-like consistency. The final paste was easier to manipulate, which could favor its use in clinical situations. In addition, Aloe Vera presents therapeutic actions [21-23] and is a water-soluble vehicle with viscosity characteristics. Even though the biocompatibility of this vehicle associated with MTA and PC has yet to be investigated, the results of the present study demonstrate that the use of Aloe Vera as a vehicle did not affect the physicochemical properties of MTA and PC. 

**Table 1:** Materials and compositions investigated

**Cement**	**Composition**	**Manufacturers**
**White structural Portland**	White clinker (100-75%), gypsum (3%), and carbonate material (0-25%)	Votorantim, SP, Brazil
**White non-structural Portland**	White clinker (74-50%), gypsum (3%), and carbonate material (26-50%)	Votorantim, SP, Brazil
**MTA BiO**	Portland cement (80%) and bismuth oxide (20%)	Angelus Ind. Prod., PR, Brazil
**ProRoot MTA**	Portland cement (75%), bismuth oxide (20%), and gypsum (5%)	Dentsply Tulsa Dental, OK, USA

**Table 2:** Physicochemical properties of the materials tested

**Test Material**	**Liquid-powder ratio**	**Setting time (min)**	**Solubility (%)**	**Dimensional change**	**pH**	**Radiopacity (mm Al)**
**Structural PC**	3.14±0.21^a^*	85.75±2.87^b^	2.30±0.36^c^	0.77±0.03^c^	11.96±0.31^a^	29.04±2.75^a^
**Non-structural PC**	3.48±0.23^a^	84.00±3.16^b^	2.81±0.40^c^	1.01±0.01^d^	11.80±0.33^a^	31.41±4.08^a^
**MTA BiO**	3.52±0.14^a^	66.75±1.71^a^	1.45±0.07^a^	0.51±0.02^b^	12.07±0.33^a^	41.49±2.20^b^
**ProRoot MTA**	3.41±0.18^a^	116.50±4.08^d^	1.74±0.13^b^	0.26±0.01^a^	11.77±0.39^a^	41.79±2.20^b^
**Control**	3.00±0.00^b^	107.50±3.21^c^	1.67±0.14^b^	0.23±0.02^a^	11.83±0.57^a^	39.88±1.97^b^

* Different letters within columns indicate statistically significant differences between the groups

Due to the lack of specific standards to test the physical properties of retrofilling materials, studies conducted in this field have used the ANSI/ADA specification no. 57 [26] as reference for endodontic sealing materials [19, 28] and the ISO 6876 specification as reference for zinc oxide and eugenol endodontic sealing materials [2]. In the clinical setting, both retrofilling and root filling materials remain in close contact with periodontal tissues, thus the ANSI/ADA standard was considered to be applicable to the materials under investigation [6, 30], following the modifications proposed by Carvalho-Júnior *et al.* [31].

The physicochemical characteristics of MTA are influenced by several factors: particle size, temperature and humidity during application, amount of air trapped in the mixture, the mixing procedure itself, and the liquid-powder ratio [3]. Determining the exact amount of powder that should be mixed with a given volume of liquid is paramount to ensure maintenance of the physicochemical properties of a material. MTA-based cements are mixed with sterile water at a 3:1 ratio according to the manufacturer’s instructions. PC, in turn, was designed for civil engineering, and a specific ratio for its use in dentistry has not been determined. In many studies, PC preparation has followed the same ratio adopted for MTA-based cements [9, 12]. However, in some applications, the liquid-powder ratio of 3:1 yields a very fluid mix, of soup-like consistency, hindering application [32]. In the present study, the mean liquid-powder ratio varied across the groups, from 3.14 to 3.52 g of powder for 1 mL of 2% glycolic solution containing Aloe Vera, showing that the amount of powder in the same bulk of water was close for different materials, *i.e.*, relatively similar regardless of the cement employed. Moreover, no statistical differences were observed for liquid-powder ratio among the cements, reinforcing that the chemical composition of MTA and PC indeed are similar [4, 9, 12, 14].

In apical surgery, root-end filling material is placed in contact with periapical tissues and is subject to washout by blood flow [29]. Therefore, a material with a shorter setting time is desirable. According to ANSI/ADA requirements [26], sealer setting time should be within 10% of the time stated by the manufacturer. In the present study, the setting time test showed significant differences between the materials tested. ProRoot MTA showed the longest setting time (116.50 min), followed by white structural PC (85.75 min) and white non-structural PC (84.00 min). The longer setting time observed for ProRoot MTA may be explained by the amount of gypsum and aluminum species present in that material [32]. Torabinejad *et al.* [2] investigated the physical and chemical properties of MTA and reported an even higher setting time than the one observed here (165 min) – a difference that may be attributed to changes in the composition of MTA powder since it was first introduced in the market [11, 19]. MTA Bio, in turn, showed the shortest setting time (66.75 min). This material consists of 80% PC and 20% bismuth oxide, and no calcium sulfate (gypsum) [8, 10]. Oliveira *et al.* [10] had already pointed out that the absence of calcium sulfate is intended to shorten the setting time reaction.

Dimensional change is another important property that needs to be carefully considered. A change in that property, possibly leading to contraction, would likely have a negative impact on the ability of MTA to seal a root end or perforation site [29]. According to ANSI/ADA standards [26], the mean linear shrinkage of a sealer should not exceed 1%, or 0.1% of expansion. In the present study, all dimensional change values were in accordance with ANSI/ADA requirements. The slight expansion observed upon setting may be explained by the absorption of water by the cements [33], and is helpful in ensuring that the seal will be present upon setting, reducing subsequent leakage [33, 34]. 

Root-end filling materials should be resistant to solubility and disintegration in an aqueous environment. According to ANSI/ADA standards [26], the solubility of sealers should not exceed 3% by mass. Solubility results obtained for all the materials tested in the present study were within ANSI/ADA recommendations (white non-structural PC 2.81%; white structural PC 2.30%; ProRoot MTA 1.74%; MTA BIO 1.45%). MTA Bio was less soluble than the other materials, which is consistent with previous findings [4, 9, 11, 34]. This difference between MTA Bio and other cements may be related to its chemical composition, which shows different structures after setting [33]. 

The biocompatibility of MTA is related to its elevated pH [19]. As a result, vehicles used in association with sealers should not adversely affect this property. In the present study, all the materials showed alkaline pH levels after being mixed with Aloe Vera, with slightly higher mean values observed for MTA Bio. MTA-based and PC cements present high amounts of calcium oxide [14], which, in contact with tissue fluid or water, are converted to calcium hydroxide. The latter is then dissociated into calcium and hydroxide ions, causing an increase in pH and calcium ion release [8, 20]. Several studies have been conducted to evaluate the pH of MTA [2, 8, 28]. pH values reported in the literature for MTA mixed with distilled water [2, 8] are lower than those found in the present study. The same was observed when MTA was mixed with propylene glycol [28]. This discrepancy could be explained by differences in study design and material composition. 

Retrofilling materials should present enough radiopacity to be radiographically distinguished from surrounding structures, *e.g.*, tooth and alveolar bone, and also to reveal empty spaces and inappropriate contours [2, 35]. According to ANSI/ADA specification no. 57 [26], an endodontic sealer material should present radiopacity correspondent to at least 3 mm aluminum. Data in Table 2 show that ProRoot MTA was the most radiopaque material (41.79±2.20), followed by MTA Bio (41.49±2.20), with no significant differences between them. Even though the two MTA-based cements contain bismuth oxide in their composition (the element responsible for radiopacity [3-6]), a lower amount of bismuth oxide has been reported for MTA Bio [6, 12]. This fact could justify the lower radiopacity of MTA Bio in comparison to ProRoot MTA. In addition, a recent study using energy dispersive x-ray microanalysis observed slight differences in bismuth oxide concentrations in gray (15.19%) *vs.* white (14.61%) MTA cements [14]. In turn, the original formulation of PC does not include bismuth oxide [3], resulting in low radiopacity and making this material difficult to be distinguished from hard tissues [36]. This would be a major disadvantage of PC in the clinical setting. In the current study, the mean values obtained for this cement were lower than 2 mm aluminum, *i.e.*, below the minimum requirement of ANSI/ADA [26]. In previous studies, the insufficient radiopacity of PC has been overcome by the addition of different radiopacifiying agents, with satisfactory results [36]. 

Even though this was an *in vitro* study, the results obtained with MTA associated with Aloe Vera are promising. It remains to be known whether the use of Aloe Vera as a vehicle would interfere with the mechanism of action of MTA, and which histological events would occur in the periapical region. Thus, further standardized *in vitro* and *in vivo* studies are indicated to check the biocompatibility, antimicrobial properties, and sealing abilities of MTA associated with Aloe Vera, before any recommendation for clinical use can be made.

## Conclusion

The physicochemical properties of the materials tested in association with Aloe Vera were compatible with ANSI/ADA requirements, except for white Portland cements, which failed to meet the radiopacity specification.
